# IKK1 aggravates ischemia–reperfusion kidney injury by promoting the differentiation of effector T cells

**DOI:** 10.1007/s00018-023-04763-2

**Published:** 2023-04-19

**Authors:** Ning Song, Yang Xu, Hans-Joachim Paust, Ulf Panzer, Maria Mercedes de las Noriega, Linlin Guo, Thomas Renné, Jiabin Huang, Xianglin Meng, Mingyan Zhao, Friedrich Thaiss

**Affiliations:** 1 Department of Critical Care Medicine, The First Affiliated Hospital of Harbin Medical University, Youzheng St 23, Harbin, 150001 China; 2 III. Department of Medicine, University Medical Center Hamburg-Eppendorf, Martinistraße 52, Hamburg, 20246 Germany; 3 Department of General, Visceral and Thoracic Surgery, University Medical Center Hamburg-Eppendorf, Hamburg, 20246 Germany; 4 Department of Pathology, Renal Pathology, University Medical Center Hamburg-Eppendorf, Hamburg, 20246 Germany; 5 Institute for Clinical Chemistry and Laboratory Medicine, University Medical Center Hamburg-Eppendorf, Hamburg, 20246 Germany; 6 Irish Centre for Vascular Biology, School of Pharmacy and Biomolecular Sciences, Royal College of Surgeons in Ireland, Dublin, Ireland; 7 Center for Thrombosis and Hemostasis (CTH), Johannes Gutenberg University Medical Center, Mainz, 55131 Germany; 8 Institute for Medical Microbiology, Virology and Hygiene, University Medical Center Hamburg-Eppendorf, Hamburg, 20246 Germany; 9 Cancer Institute, Fudan University Shanghai Cancer Center, Shanghai, 200032 People’s Republic of China; 10 Heilongjiang Provincial Key Laboratory of Critical Care Medicine, The First Affiliated Hospital of Harbin Medical University, Harbin, 150001 China

**Keywords:** AKI: acute kidney injury, Non-canonical NFκB, IKKalpha, CD4^+^T cells, Th17 lymphocytes

## Abstract

Ischemia–reperfusion injury (IRI) is one of the major causes of acute kidney injury (AKI), and experimental work has revealed detailed insight into the inflammatory response in the kidney. T cells and NFκB pathway play an important role in IRI. Therefore, we examined the regulatory role and mechanisms of IkappaB kinase 1 (IKK1) in CD4^+^T lymphocytes in an experimental model of IRI. IRI was induced in CD4cre and CD4IKK1Δ mice. Compared to control mice, conditional deficiency of IKK1 in CD4^+^T lymphocyte significantly decreased serum creatinine, blood urea nitrogen (BUN) level, and renal tubular injury score. Mechanistically, lack in IKK1 in CD4^+^T lymphocytes reduced the ability of CD4 lymphocytes to differentiate into Th1/Th17 cells. Similar to IKK1 gene ablation, pharmacological inhibition of IKK also protected mice from IRI. Together, lymphocyte IKK1 plays a pivotal role in IRI by promoting T cells differentiation into Th1/Th17 and targeting lymphocyte IKK1 may be a novel therapeutic strategy for IRI.

**Supplementary Information:**

The online version contains supplementary material available at 10.1007/s00018-023-04763-2.

## Introduction

Acute kidney injury (AKI) is a systemic disease without effective therapy which significantly increases patient morbidity and mortality. Ischemia–reperfusion injury (IRI) is one of the major causes of AKI. IRI occurs frequently due to a transient reduction or cessation of blood flow, followed by reperfusion during the perioperative period or in septic disease or after kidney transplantation [1]. Recent research efforts have shown that both innate and adaptive immune responses mediate renal damage in IRI-induced injury [2].

T cells, especially CD4^+^T cells, have been demonstrated to play an important role in the pathophysiology of ischemic AKI [3–5]. In particularly, it has also been reported that both functional and histological kidney injuries were dependent on the production of the cytokine IL17A, and that both were attenuated by depleting IL17A [6].

NFκB functions as a homo- or heterodimer of five constitutive proteins [c-REL, RelA (p65), RelB, p50, and p52] and plays a key role in the differentiation of T helper cells [7, 8]. Under basal conditions, NFκB dimers are retained in the cytoplasm by a family of inhibitory proteins, IκBs. Canonical NFκB activation occurs via the phosphorylation of these inhibitory IκB proteins by the IκB kinase (IKK) complex [9]. The IKK complex contains three kinases: IKK1 and IKK2, and a regulatory kinase termed NFκB essential modulator (NEMO) [10]. The non-canonical NFκB pathway activation depends on NFκB-inducing kinase (NIK), which integrates signals from cell receptor and activates IKK1, for triggering p100 phosphorylation and processing of the p52/RelB NFκB complex [11]. The potential clinical importance of NFκB as a therapeutic target in treating IRI has been derived from several experimental studies that demonstrated the amelioration of IRI after the inhibition of NFκB pathway activation [12–14].

We have shown previously that lymphocyte-specific deletion of IKK2 or NEMO mediates an increase in intrarenal Th17 cells and accelerates renal damage in an IRI mouse model [15]. We therefore here address the role of the IKK-kinases in CD4^+^T cells by conditional deletion of IKK1, NIK, IKK2 and NEMO. To target NFκB pathway activation selectively in CD4^+^T cells, we crossed CD4cre mice with IKK1flox, IKK2flox, NEMOflox and NIKflox animals and challenged animals in a kidney IRI model. Our results demonstrate that NFκB pathway is activated in kidney including renal CD4^+^T cells after IRI. Inhibition of IKK1 in CD4^+^T cells significantly reduced renal damage 2d after IRI induction and impaired T cells differentiation into Th1/Th17 cells as shown by single cell sequencing data analysis of lymphocytes isolated from kidneys after IRI induction. Therefore, inhibition of IKK1 selectively in CD4^+^T cells might be an interesting candidate to be further examined as a novel therapeutic tool for interference with IRI.

## Material and methods

### Animals and animal model

Mice expressing Cre recombinase under the CD4 promoter were crossed with floxed IKK1 [16], IKK2 [17, 18], NEMO [19] or NIK [20] mice. The mice were generously provided by Dr. M. Karin (San Diego) for IKK1fl/fl and IKK2fl/fl mice, Dr. C. Wilson (Seattle) for CD4cre mice, Dr. M. Pasparakis (Cologne) for NEMOfl/fl mice and Dr. N. Ghilardi (Genentech) for NIKfl/fl mice. All animals were backcrossed to C57BL/6 wild type mice for more than 10 generations and housed at the animal facility of the University Medical Center Hamburg-Eppendorf. Animal experiments were performed according to national and institutional animal care and ethical guidelines and were approved by local committees (permission number G25/17).

8–12 weeks old male mice were anesthetized with isoflurane, buprenorphine subcutaneous and metamizole oral were administered for pain control. After placing the animals on a heated operation pad, the left and right renal pedicles were identified through an abdominal midline incision and occluded with atraumatic microvascular clamps for 45 min. Then, the clamps were released, and the kidneys allowed reperfusion. After visible reperfusion was completed, the abdomen was closed with continuous sutures, as previously described [15]. Technical details which have to be taken into account in the IRI disease models have been described recently by others and were all considered for our experiments [21]. Sham-operated animals were subjected to the same operating procedure without clamping. During the procedure, the animals were hydrated with saline. During experiments, mice were monitored daily and mice with signs of severe disease were euthanized with an O2/CO2 mixture. All experimental procedures were performed during the same time period and all groups were examined in parallel to minimize group and mice strain variabilities. The animals were sacrificed 2 days after IRI. In each group, at least five animals were examined, and the experiments were repeated three times. The inhibitor KINK1, also known as Bay 65–194, was kindly provided by Dr. K. Ziegelbauer (Bayer Health Care, Berlin, Germany). The inhibitor was dissolved in 10% Cremophor and injected intraperitoneally in an amount of 5 mg/kg body weight.

### Renal functional studies and histology

BUN and serum creatinine were analyzed using Siemens Healthcare (Erlangen, Germany) Atellica Solution analyzers and reagents. Kidney sections were fixed in buffered formalin for 24 h, processed and embedded in paraffin wax. Tubular injury was assessed on periodic acid-Schiff–stained sections. The tubular injury index, determined by assessing tubular epithelial cell loss, tubular necrosis, accumulation of cellular debris, and tubular cast formation, was scored according to the percentage of affected tubules under high-power microscopy. The percentage of tubules affected was assigned a score: 0, normal; 1, 10–25%; 2, 26–50%; 3, 51–75%; and 4, 75%, according to Chan et al.[6]. Tissue samples were counted in a blinded manner.

### Flow cytometry

Cell isolation and staining were performed as recently published [22]. For intracellular cytokine staining, cells were activated by incubation for 3 h with PMA (50 ng/mL; Sigma-Aldrich, Germany), brefeldin A (5 μg/mL; Sigma-Aldrich) and ionomycin (1 μg/mL; Calbiochem-Merck, Germany). Fluorochrome-labeled antibodies against IL17A, IFNγ (BD Biosciences, Heidelberg, Germany) and commercial Perm/Wash buffer (BD Biosciences) were used after stimulation. Dead cells were excluded from the analysis using Near-IR Live/Dead fixable staining reagent (ThermoFisher). Cells in all FACS plots were pre-gated based on CD45 expression. Measurement was performed on a BD LSRII Cytometer.

### Quantitative real-time PCR analysis

Total RNA of the renal cortex was prepared, and real-time PCR was performed according to standard laboratory methods. Real-time PCR was performed for 40 cycles on a QuantStudio 3 PCR systems (Thermo Fisher, Waltham, MA) with the 18S rRNA as a housekeeping gene.

### RNA isolation from renal CD4^+^ T cell and bulk RNA sequencing

Renal CD4^+^ T cell were enriched with CD4^+^ T cell isolation kit (Miltenyi Biotec) in accordance with the manufacturer’s instructions and CD4^+^ T cells were further purified by FACS ARIA sorting. RNA of CD4^+^ T cells (≤ 5 × 10^4^ cells) was extracted by RNeasy micro kit 50 (QIAGEN, Germany). Samples that passed quality control were amplified with the SMARTer kit and then subjected to second generation sequencing via the Illumina platform. The sequenced results were quality controlled in the using the fastqc package. Samples that passed quality control were removed using the Trimmomatic package to remove poor quality sequences and artificially added plug sequences during sequencing. A mean of 39,039,302 paired-end, 150 nt reads were generated. Bases less than Q30 and adapter sequences of the reads were trimmed, and any reads shorter than 36 nt were removed using Trimmomatic version 0.36 [23]. The trimmed sequencing results which passed quality control reads were then aligned to the mice reference assembly GRCm39 with “Kallisto” analyzed with “DE-seq2” [24]. Normalization and differential expression analyses were performed with DESeq2 [25].

### Single cell RNA sequencing analysis

The Seurat package (version 4.0.2) was used to perform unsupervised clustering analysis on scRNA-seq data. In brief, gene counts for cells were normalized by library size and log transformed. To reduce batch effects, we apply the integration method implemented in the latest Seurat version 4 (function FindIntegrationAnchors and IntegrateData, dims = 1:30). The integrated matrix was then scaled by ScaleData function (default parameters). Principal component analysis was performed on the scaled data (function RunPCA, npcs = 30) to reduce dimensionality. 30 principal components were determined using the ElbowPlot function and used to compute the KNN graph based on the euclidean distance (function FindNeighbors), which then generated cell clusters using function FindClusters. Uniform Manifold Approximation and Projection (UMAP) was used to visualize clustering results. The top differential expressed genes in each cluster were found using the FindAllMarkers function (min.pct = 0.1) and ran Wilcoxon rank sum tests. The differential expression between clusters was calculated by FindMarkers function (min.pct = 0.1), which also ran Wilcoxon rank sum tests.

### NFκB pathway activation score

A *NFκB pathway activation score* was calculated by selection of 9 genes related to NFκB -pathway activation according to the literature published: *Nfkb1, Nfkb2, Rela, Relb, Rel, Chuk, Ikbkb, Ikbkg, Map3k14*. For bulk sequencing, the *NFκB pathway activation score* was generated by transforming log2 expression profiles into Z-scores, and averaging Z-scores of the NFκB related gene sets to generate a tissue score for each sample.

### Statistics

Statistical analysis was performed using GraphPad Prism (La Jolla, CA). The results are shown as mean ± SEM when presented as a bar graph or as single data points with the mean in a scatter dot plot. Differences between two individual groups were compared using Mann–Whiteney test. In the case of three or more groups, a one-way ANOVA with Holm-Sidak’s multiple comparisons test was used.

## Results

### NFκB pathway activation in the kidney after IRI

**Fig. 1:**
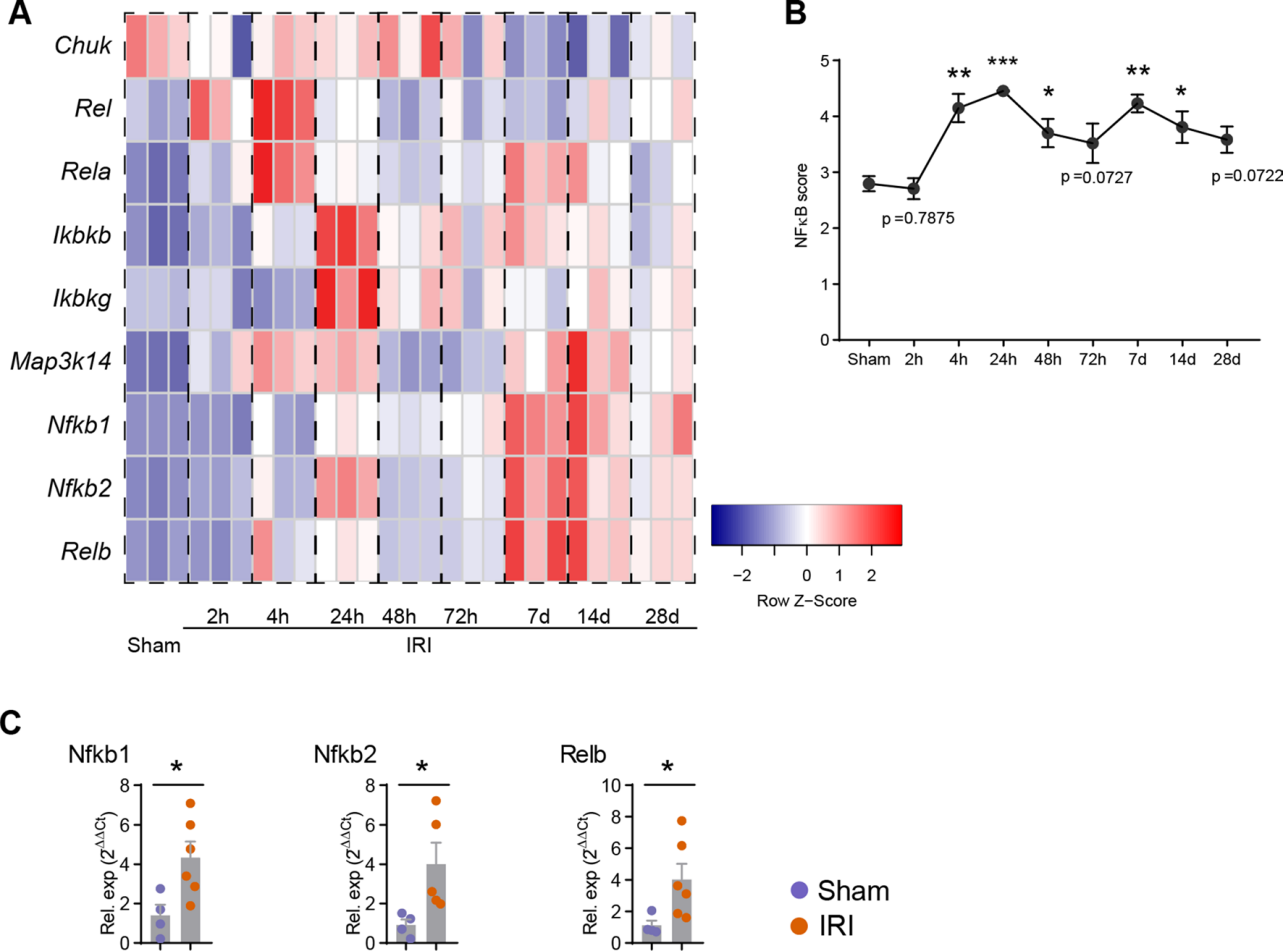
Enrichment of NFκB signaling in kidney during ischemia–reperfusion induced kidney injury. **A** NFκB inflammatory pathway activation in kidney samples of sham-operated mice and of mice after ischemia–reperfusion at 2h, 4h, 24h, 48h, 72h, 7d, 14d and 28d. **B** Quantification of the NFκB inflammatory pathway based on cumulative gene expression in the respective groups. **C** RT-PCR analysis of renal *Nfkb1*, *Nfkb2* and *Relb* expression in control and after IRI-induced (48h) mice. (*P < 0.05, ***P < 0.005, ****P < 0.001)

To verify the activation of NFκB pathway in kidney during IRI, we interrogated bulk RNA dataset (GSE98622) [26] of mouse kidneys, collected at the timpoints of 2h, 4h, 24h, 48h, 72h, 7d, 14d and 28d after IRI as well as corresponding control renal RNA from sham-operated mice for the expression levels of 9 NFκB-activation genes (*NFκB activation score*). NFκB-activation score was upregulated at all timepoints beyond 2h after IRI induction compared to sham-operated group (Fig. 1A, B). Moreover, when we performed RT-PCR analysis of *Nfkb1*, *Nfkb2* and *Relb* expression in kidneys after IRI, expression of these genes also significantly increased after IRI induction (Fig. 1C).

**Fig. 2:**
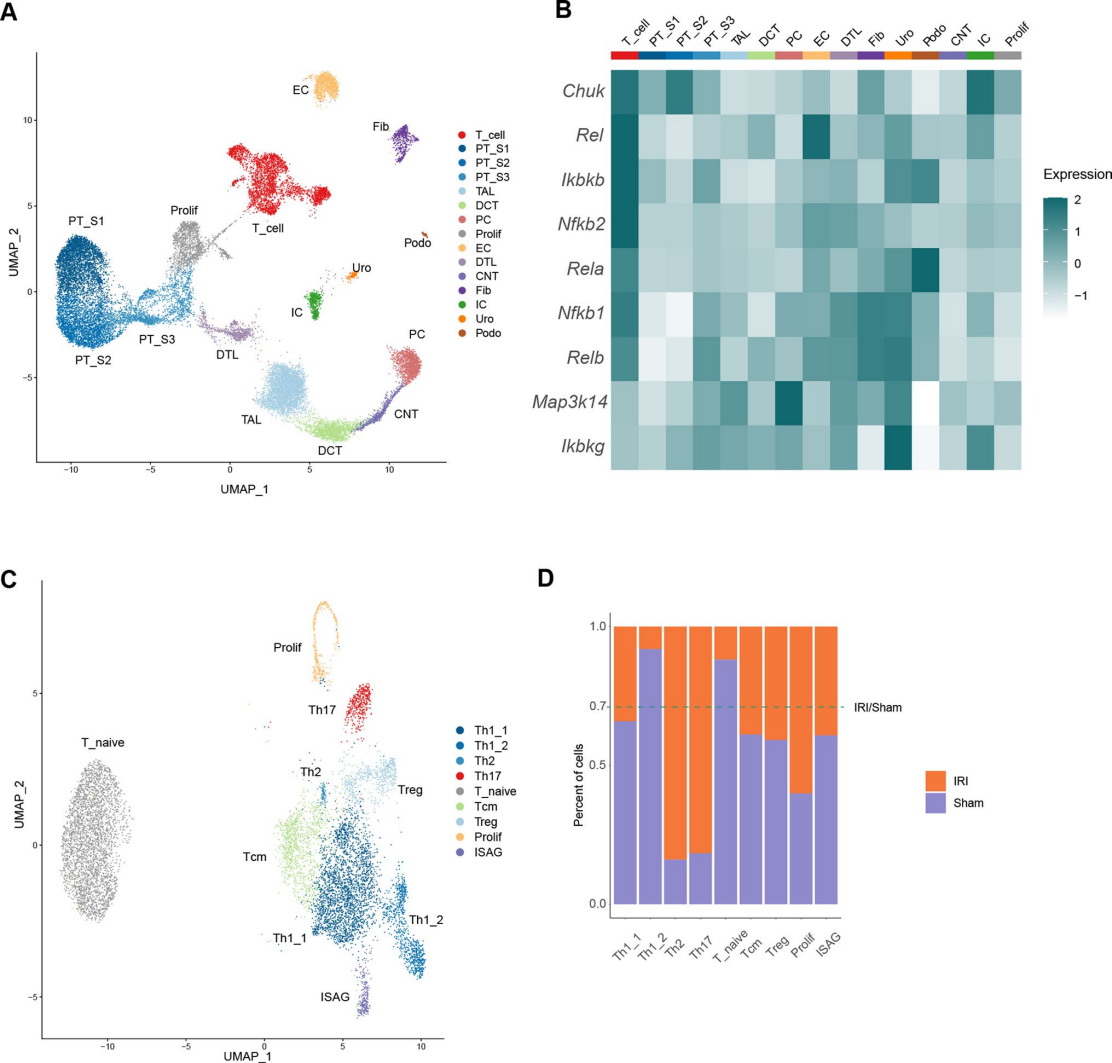
Enrichment of NFκB signaling in renal CD4^+^T cells during ischemia–reperfusion induced kidney injury. **A** Clustering and UMAP dimensional reduction of integrated sn/scRNA-seq from wildtype mice 2 days after IRI. Clusters were annotated according to their gene expression profiles. **B** Quantification of the NFκB score based on cumulative gene expression in the respective cell population (see also supplementary Fig. 1). **C** Clustering and UMAP dimensional reduction of scRNA-seq of renal CD4^+^ T cells from sham-operated and IRI induced mice. Clusters were annotated according to their gene expression profiles. **D** Fraction of cells in the respective clusters from sham-operated and IRI induced mice

To capture kidney cells and renal CD4^+^T cells landscape of IRI, we integrated online single nuclear RNA sequencing dataset of whole mouse kidney (GSE139107) [27] and single cell RNA sequencing dataset of CD4^+^T cells (E-MTAB-8002) [28] 2 days after IRI (Fig. 2A). Unsupervised clustering recognized 14 renal clusters and 1 CD4^+^T cell cluster including both epithelial (descending loop of Henle, thin ascending limb) and nonepithelial (immune, endothelial, stromal) cells in the kidney after IRI induction. Based on the gene markers provided by the original published articles [27], we identified the following cell populations: PT-S1, S1 segment of proximal tubule; PT-S2, S2 segment of proximal tubule; PT-S3, S3 segment of proximal tubule; TAL, thick ascending limb of loop of Henle; DCT, distal convoluted tubule; PC, principal cells of collecting duct; Prolif, proliferating cells; EC, endothelial cells; DTL, descending limb of loop of Henle; CNT, connecting tubule; Fib, fibroblasts; IC, intercalated cells of collecting duct; Uro, urothelium; Pod, podocytes and T cells, CD4^+^T cells (Fig. 2A, Supplemental Table 1). We evaluate the NFκB activation score in all cell populations and found that this score was significantly upregulated in T cells when compared with all the cell populations (Figs. 2B and S1). Next, we analyzed more precisely the CD4^+^T cells dataset 2 days after IRI (E-MTAB-8002) and integrated with CD4^+^T cells from control mice. With unsupervised clustering, we identified 9 cell populations, including Th1_1, Th1_2, Th2, Th17, Tnaive (naïve T cells), Tcm (central memory T cells), Treg (regulatory T cells), Prolif (Proliferating T cells) and ISAG (IFN signaling–associated gene highly expressed T cells) (Fig. 2C, supplemental Table 2). To dissect the effect of IRI on the CD4^+^T cells composition in the kidney, we compared subclusters of CD4^+^T cells from control mice and IRI mice (Fig. 2D). The results demonstrated that the population of Th17 was more abundant in the inflamed kidney, indicating that this population could be an important pathogenic factor during early phase of IRI. Taken together, this prompted us to hypothesize that NFκB activation in CD4^+^T cells contributes to the pathogenesis of IRI.

### CD4^+^T cell-derived IKK1 drives IRI

**Fig. 3:**
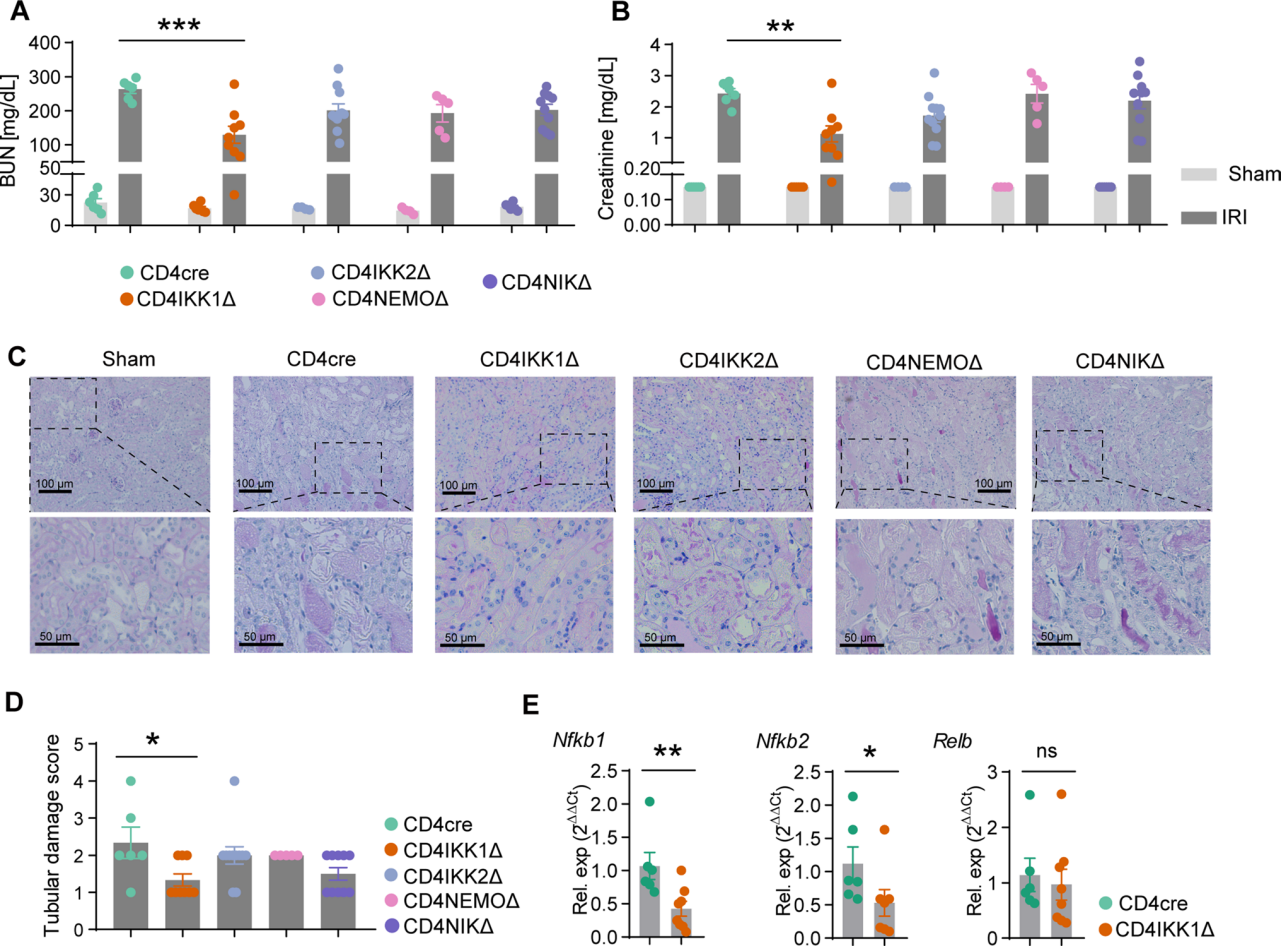
Inhibition of IKK1 in CD4^+^ T cells protect mice from kidney injury. **A** Quantification of BUN concentration from CD4cre, CD4IKK1Δ, CD4IKK2Δ, CD4NEMOΔ, CD4NIKΔ mice 2 days after IRI induction and corresponding sham-operated mice. **B** Quantification of blood creatinine concentration. **C** Representative photographs of PAS-stained kidney sections from sham-operated, CD4cre, CD4IKK1Δ, CD4IKK2Δ, CD4NEMOΔ, and CD4NIKΔ mice 2 days after IRI induction. **D** Tubulointerstitial damage score.Symbols represent individual data points with the mean as a bar. **E** RT-PCR analysis of renal *Nfkb1*, *Nfkb2* and *Relb* expression in CD4cre or CD4IKK1Δ mice. (*P < 0.05, **P < 0.01, ***P < 0.005, ****P < 0.001)

To investigate the functional role of NFκB activation in CD4^+^T cells during kidney inflammation, we specifically deleted IKK1(*Chuk*), IKK2(*Ikbkb*), NEMO(*Ikbkg*) and NIK(*Map3k14*) which are the key molecules that activate NFκB pathways in CD4^+^T cells by crossing CD4cre with corresponding flox mice. Blood urea nitrogen (BUN) levels and Creatinine which are representative markers of functional kidney impairment, were significantly reduced in CD4IKK1Δ mice compared to control mice, whereas there is no significant difference in CD4IKK2Δ, CD4NEMOΔ and CD4NIKΔ mice (Fig. 3A, B). Moreover, the morphological evaluation of kidney sections also revealed a significant reduction of renal tubular damage in CD4IKK1Δ mice but not in the other groups (Fig. 3C, D). Additionally, gene expression of renal *Nfkb1*and *Nfkb2* was also significantly reduced in CD4IKK1Δ mice, which demonstrates that the activation of NFκB pathway is significantly changed in CD4IKK1Δ mice (Fig. 3E). In conclusion, our data show that CD4^+^T cell derived IKK1 is a major driver of renal tissue injury in IRI.

### Pharmacological inhibition of IKK1 protect mice from IRI

**Fig. 4:**
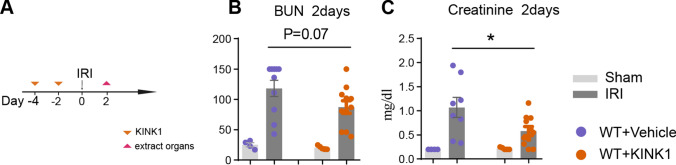
Pharmacological inhibition of IKK1 protected mice from IRI. **A** IRI was induced in wildtype mice and inhibitor KINK1 was given by intraperitoneally injection at indicated time points. **B** Quantification of BUN levels in serum from mice which received vehicle or KINK1 2 days after IRI induction and corresponding sham-operated mice. **C** Quantification of creatinine levels in serum. Symbols represent individual data points with the mean as a bar (*P < 0.05, **P < 0.01, ***P < 0.005, ****P < 0.001)

With respect to the IKK1 pathogenic role in IRI and its importance for the CD4^+^T cells differentiation process, we next investigated as to whether pharmacological block of IKK1 could alleviate kidney damage. Therefore, we blocked IKK1 activation by treating mice with KINK-1 [29], a IKK1/IKK2 inhibitor, before and after the induction of IRI (Fig. 4A). Upon induction of IRI, IKK1 inhibition reduced the severity of renal damage compared to vehicle treatment (Fig. 4B), consistent with our earlier reported data [15].

### IKK1 promotes cytokines production of CD4^+^T cells

**Fig. 5:**
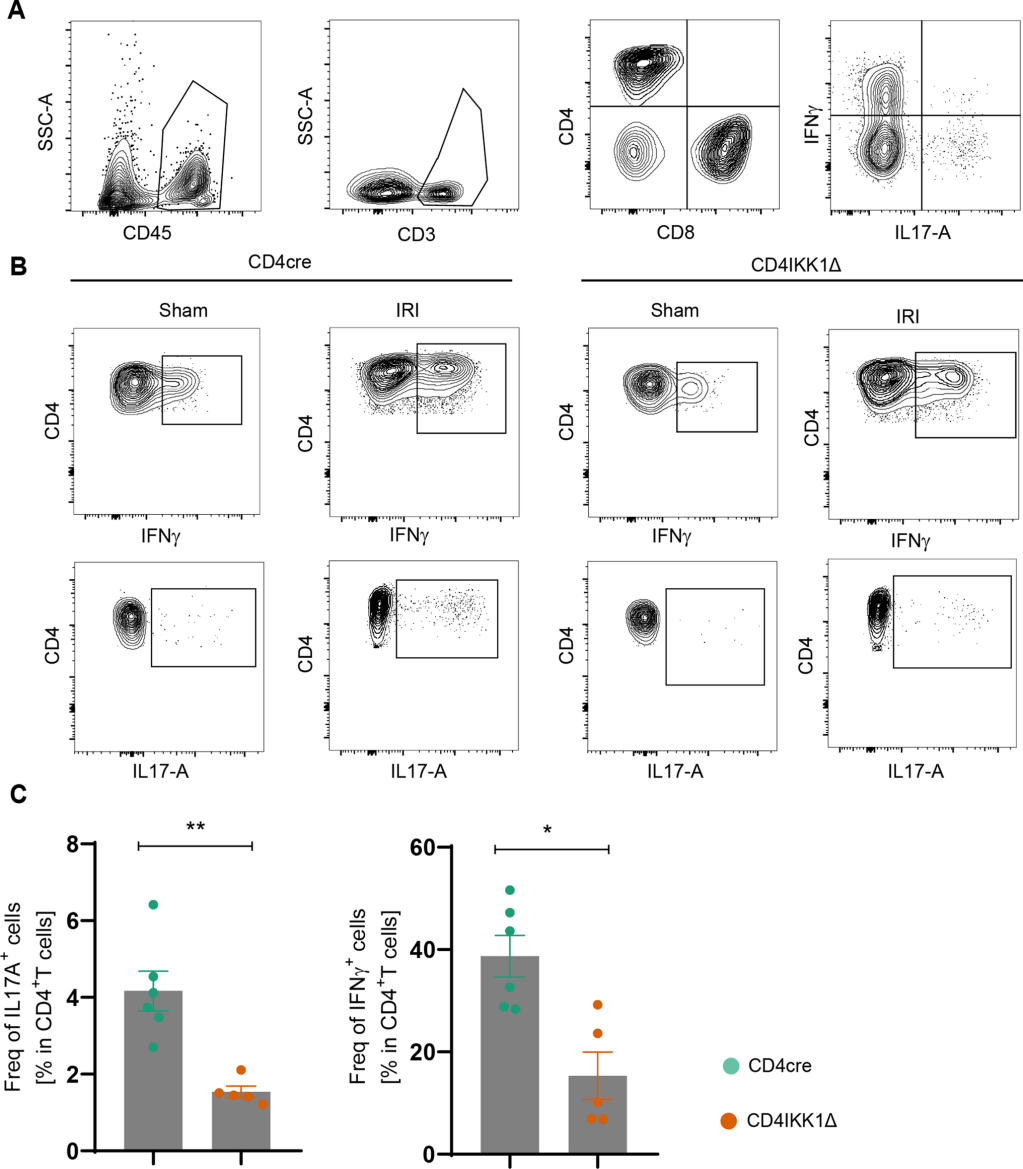
Deficiency of IKK1 in CD4^+^ T cells suppresses cytokines production during IRI. **A** Gating strategy of infiltrated renal lymphocytes. **B** Representative FACS data of indicated cytokines produced in renal CD4^+^ T cells after stimulation with PMA/ionomycin. **C** Quantification of indicated cytokines produced in renal CD4^+^T cells from CD4cre and CD4IKK1Δ mice. Symbols represent individual data points with the mean as a bar (*P < 0.05, **P < 0.01, ***P < 0.005, ****P < 0.001)

To investigate the effect of IKK1 on the cytokine production of renal CD4^+^T cells after IRI, we performed flow cytometry of immune cells infiltrating the kidneys. Flow cytometry-based staining of leukocytes from CD4cre and CD4IKK1Δ mice revealed that about 40% of lymphocytes infiltrated in kidney are CD4^+^T cells 2 days after IRI induction (Fig. 5A). Cytokine staining results highlighted that both IFN-γ and IL-17A production increased after IRI induction, furthermore, both cytokines production significantly decreased in CD4IKK1Δ mice comparing to CD4cre mice (Fig. 5B, C). In conclusion, flow cytometry results confirmed the enrichment results of RNA transcriptome, in which IKK1 has a major pathogenic role in the differentiation of Th1 cells and Th17 cells.

In contrast, in CD4NIKΔ mice the number of IFN-γ and IL-17A producing CD4^+^T cells in kidneys was not reduced when compared with CD4cre animals after IRI induction (Fig. S2).

### IKK1 drives Th17 and Th1/Th2 differentiation of CD4^+^T cells

**Fig. 6:**
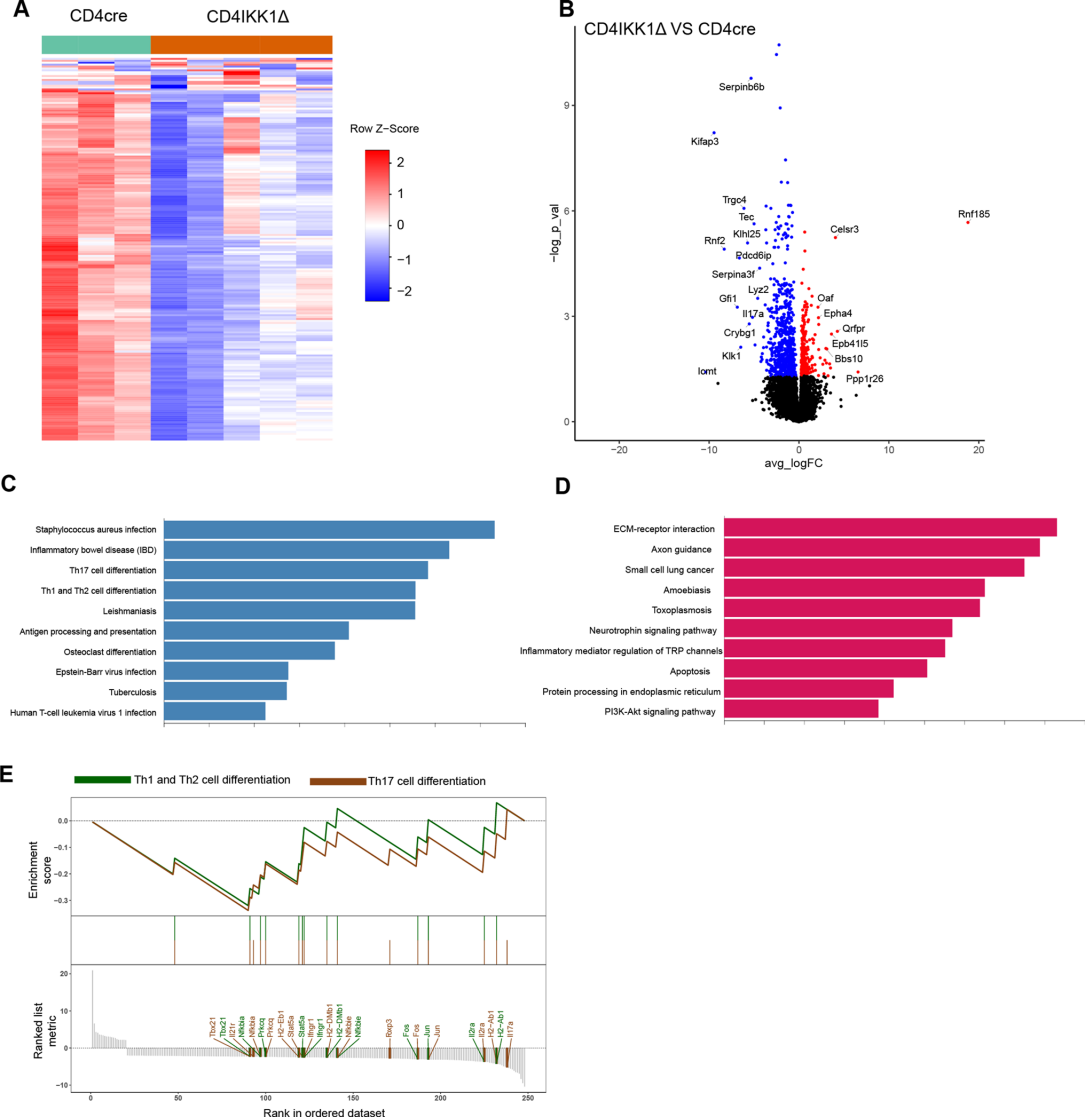
IKK1 drive pathogenesis of CD4^+^ T cells during IRI. **A** Heatmap shows 200 random genes expressed by renal CD4^+^ T cells from CD4cre (*N* = 3) and CD4IKK1Δ (*N* = 5) mice on day 2 after IRI. **B** Volcano plot shows differentially expressed genes (DEG) between CD4^+^ T cells from CD4cre and CD4IKK1Δ(blue: downregulated in CD4IKK1Δ; red: upregulated in CD4IKK1Δ; black: adjusted p value less than 0.05). **C** Bar plot shows top 10 enriched KEGG pathways of downregulated genes in CD4 + Tcells of CD4IKK1Δ mice. **D** Bar plot shows top 10 enriched KEGG pathways of upregulated genes in CD4 + T cells of CD4IKK1Δ mice. **E** GSEA enrichment plot shows enriched genes of Th1 and Th17 differentiation pathway

To further explore the mechanism underlying for the pathogenic role of lymphoid IKK1 in IRI, we performed bulk RNA sequencing to analyze RNA transcriptome profiles of renal CD4^+^T cells from CD4cre or CD4IKK1Δ mice after IRI. Individual replicate in each group were highly homologous according to their gene expression profiles and overall expression similarity (Fig. 6A). There were in total 248 DEG (FDR < 0.05, and > twofold differences in expression) recognized, including 228 down-regulated genes (for example Il17a, Il2ra), and 20 up-regulated genes (Celsr3, Rnf185) (Fig. 6B). Among down-regulated genes, most of them were inflammation related and enriched with KEGG (Kyoto Encyclopedia of Genes and Genomes) terms “Th1 and Th2 cell differentiation”, “Th17 cell differentiation” and “inflammatory bowel disease” (Fig. 6C), whereas up-regulated genes were mostly enriched with “ECM-receptor interaction”, “Apoptosis” and “Axon guidance” (Fig. 6D). The enrichment of *Tbx21*, *Il2ra*, *Il17a* and *H2-Ab1* in “Th1/Th2 cell differentiation” and “Th17 cell differentiation” were also in line with the understanding of how NFκB regulate T cell immune response (Fig. 6E).

**Fig. 7:**
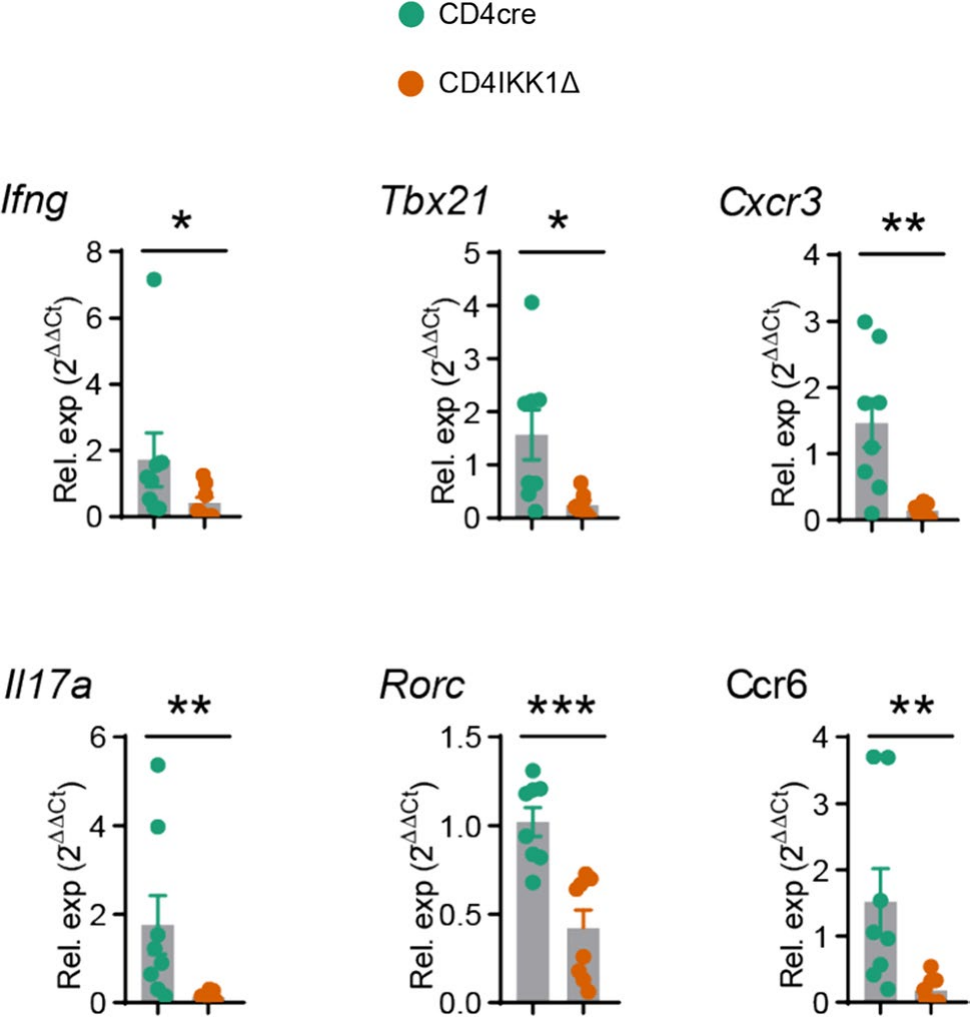
Deficiency of IKK1 in CD4^+^ T cells inhibit differentiation of effector T cells after IRI. RT-PCR analysis of renal *Ifng, Tbx21, Cxcr3, Il17, Rorc* and *Ccr6* expression in CD4cre or CD4IKK1Δ mice. (*P < 0.05, **P < 0.01, ***P < 0.005, ****P < 0.001)

RT-PCR analysis of renal *Ifng*, *Tbx21*, *Cxcr3*, *Il17*, *Rorc* and *Ccr6* gene expression also confirmed that the differentiation of Th1 and Th17 was dependent on IKK1 in the inflamed kidneys after IRI (Fig. 7).

Contrary to the results in CD4IKK1Δ mice, specifically deletion of NIK in CD4^+^ T cells did not inhibit differentiation of Th1/ Th17 cells after IRI-induction (Fig. S3).

In conclusion, IKK1 plays a significant pathogenic role in IRI by affecting the differentiation of CD4^+^T cells, and therapeutic inhibition of IKK1 specifically in CD4^+^T cells could reduce IRI damage.

## Discussion

The activation of NFκB plays a pivotal role in renal injury after IRI as we and others have shown previously [9, 15, 30]. NFκB is activated both in renal parenchymal and in infiltrating inflammatory cells after IRI-induction [31–35]. Therefore, in the current experiments using RNA sequencing analysis we now confirmed that (1) NFκB pathway is activated up to 28d after induction of IRI (Fig. 1) and (2) NFκB is activated both in renal parenchymal cells and infiltrating T cells (Fig. 2).

NFκB activation is induced by two major pathways, the canonical pathway with the kinase complex including NEMO-IKK2-IKK1 kinases and the non-canonical pathway with the NIK-IKK1 kinase complexes respectively [36–38]. To get further insight into the potential role of NFκB activation selectively in CD4^+^T cells after IRI-induction, we therefore specifically ablated IKK1, IKK2, NEMO, and NIK in CD4^+^ T cells via crossing CD4cre mice with the corresponding kinase-floxed animals. Lymphocyte-specific inhibiting of IKK1, but not IKK2, NEMO or NIK in CD4^+^ T cells protected mice from IRI (Fig. 3).

To the best of our knowledge these are the first experiments which demonstrate that specific inhibition of IKK1 selectively in CD4^+^ T cells had profound effects on kidney function and morphology after IRI-induction and further addresses the role of NFκB kinases in lymphocytes after renal IRI-injury we described previously [15]. It has been shown previously by others that inhibition of NFκB activation in renal tubular epithelial cells [33] and T cells using a super repressor form of an IkBα transgene [39] protected mice from IRI injury.

NFκB pathway activation controls the development of T cells and the differentiation of Th1, Th17 and Tregs [8, 40, 41]. It has been shown recently that Tregs, which have an inflammatory suppressive function, play an important role in the late phase of IRI [28]. We therefore focused on the early time points after IRI-induction and further addressed the question whether inhibition of NFκB pathway activation specifically in T cells could reduce the extent of IRI disease at 48 h after IRI-induction by affecting the differentiation of pro-inflammatory cells into Th1 and Th17 cells to further extend data published by us and others previously [42, 43].

Next, to examine possible treatment approaches in this IRI-model through IKK1 inhibition, animals were treated with KINK1 [29]. We could confirm our previously published data that systemic application of high concentration of KINK1 improved kidney function as shown by decreased BUN and creatinine serum levels (Fig. 4) [15]. This in vivo inhibition of IKK-activation is of course not T-cell specific and may also be due to reduced IKK-activation in tubular epithelial cells after IRI-induction. But it gives some preliminary insight that IKK1 might be an interesting candidate to be examined further as a treatment option in the IRI-model and other models of inflammatory renal disease. For clinical trials more specific IKK1-inhibitors are needed such as those now being explored to treat cancer [44, 45] and it has also to be proven first experimentally that treatment with a specific IKK1-inhibitor also reduces injury in the IRI-model even when given with some delay after the disease was established.

Next, we examined whether functional and morphological changes in control animals and mice with lymphocyte specific IKK1 deletion alters lymphocyte infiltration into kidneys by FACS analysis of CD4^+^T cells infiltrating kidneys 48 h after IRI-induction (Fig. 5). Inhibition of IKK1-activation selectively in CD4^+^T cells significantly reduced the percentage of IL17A and IFN-γ infiltrating cells in kidneys after IRI-induction which further supports the pathogenic role of IKK1 in T cells in this IRI model.

The pathogenetic role of Th17-cells in immune-mediated glomerular diseases and ischemia- reperfusion injury has been demonstrated recently by us and others [15, 22, 46–49]. The exact molecular mechanisms how Th17-cells are regulated once infiltrating into the kidney and how Th17-cells induce kidney injury by attacking endogenous renal glomerular, tubular epithelial and vascular endothelial cells or kidney fibroblasts remains to be defined [50–52].

To get further insight into the relevant mechanisms, we next isolated CD4^+^-lymphocytes from kidneys 48 h after IRI induction and single cell RNA sequencing was performed. Isolating CD4-positive T cells from sham-operated mice and animals 48 h after IRI-induction was unique in our experimental procedure when compared with similar data published by other groups which performed single cell sequencing from whole kidney extracts. This procedure allowed to further characterize the transcriptome profile of kidney infiltrating CD4^+^ T cells. Using this approach, we could convincingly demonstrate transcriptome activation in CD4^+^ T lymphocytes of CD4cre mice which was markedly reduced in CD4IKK1Δ animals (Fig. 6A). Volcano blots showed differentially expressed genes (Fig. 6B) which will allow us to further characterize the differences of T cell activation between control and IKK1-deleted CD4^+^ T cells in the IRI model.

RNA sequencing, as one of the most high-profile biological research tools in recent years, has been used extensively in IRI research [53–58]. However, RNA sequencing has its limitations as RNA sequencing results are based on the captured RNA located in the cell, and if regulation occurs at the post-transcriptional or at the protein level, then the results of RNA sequencing may not match or even reverse the true level [59, 60] which has to be taken into account when further looking in more detail into the role of IKK1-induced NFκB transcription factors activation might play in the regulation of CD4^+^T cells in kidney inflammatory diseases, in particular IRI.

We next further examined GSEA enrichment plot comparing RNA sequencing result of renal CD4^+^T cells from CD4IKK1Δ mice and CD4cre control groups which showed that enriched genes of Th1 and Th17 differentiation pathway were profoundly reduced in IKK1-deleted T cells (Fig. 6E). Mechanistically, we therefore could demonstrate that inhibition of IKK1 impaired the ability of CD4^+^T cells to differentiate towards Th1 and Th17 and thereby reduced the pathogenicity of CD4^+^T cells and improved kidney function after IRI-induction.

Our findings are in accordance with data published previously in which IKK1 has been shown to be a key transcriptional regulator of the Th17 lineage and maintained the Th17 phenotype by activating the Il17a gene [61, 62]. It is therefore reasonable to assume that inhibition of IKK1 in CD4^+^ T cells in our study reduced the severity of IRI through effecting the activation of non-canonical NFκB pathway. The fact that in our experiments no protective effect was obtained for NIK inhibition in CD4^+^T cells in this IRI model may be attributable to other unidentified, NIK-independent molecules that regulate the activation of IKK in the non-canonical NFκB pathway as for example has been shown recently that IKKα-dependent phosphorylation of S376 stimulated whereas IKKα-independent phosphorylation of S484 inhibited RORγt function in Th17 differentiation [63].

IKK1-inhibition may also affect regulation of Tregs. Inhibition of IKK1 in kidneys using shRNA-IKK1 lentiviral vectors or using conditional knockout mice (Ksp-IKKα^−/−^) in which the IKK1 gene was specifically disrupted in renal TECs before IRI-induction, renal damage was reduced through IL10-producing regulatory T cells [64]. Regulatory T cells play an important role during the resolution phase after IRI-induction [28, 65–67].

What is known about the potential therapeutic approaches targeting canonical NFκB signaling in the early inflammatory response to renal IRI has been summarized in excellent reviews recently [13]. Our data now add the non-canonical NFκB signaling via IKK1 activation as an additional potential therapeutic target in the IRI-model and therefore also extends our previously published data that lymphocyte specific IKK2 and NEMO deletion accelerates renal damage in the IRI mouse model [15].

The role NFκB pathway inhibition plays after IRI induction has also to be placed in the context of a multiplayer process in the induction and also resolution phase of the disease as many pathways of the immune response are also activated after IRI-induction [68–73]. The interaction of these pathways with the NFκB pathway has to be addressed in more detail.

Much remains to be learned about the role of non-canonical and canonical NFκB pathway inhibition by IKK1 and IKK2 in lymphocytes in renal diseases, and time dependence after disease induction and differential activation of lymphocytes subsets in immune-mediated and non-immune-mediated disease models has to be evaluated further. However, our current experiments show that IKK1-inhibition in lymphocytes might be a candidate to be evaluated further as a treatment option in kidney diseases. We and others have shown previously that the activation of T cells with suppressive function, Tregs, is critical depend on the activation of IKK2 so that the activation of IKK1 and IKK2 play critical roles in different T-cell subset activation [74–77] and therefore might be therapeutic targets to inhibit or activate different T-cell subpopulations at different time points as kidney diseases progress.

In conclusion, based on our current data, we propose that inhibition of IKK1 could impair the ability of CD4^+^T cells to differentiate towards Th1 and Th17 cells by affecting the activation of the NFκB non-classical pathway, thereby reducing the extent of IRI damage.

## Supplementary Information

Below is the link to the electronic supplementary material.

Supplementary file1 (DOCX 414 KB)

Supplementary file2 (XLSX 14 KB)

Supplementary file3 (XLSX 12 KB)

## Data Availability

All raw data are available via the Sequence Read Archive via GEO (GSE217696).
