# Supplementary material for: IKK1 aggravates ischemia–reperfusion kidney injury by promoting the differentiation of effector T cells
Source: Cell Mol Life Sci. 2023 Apr 19;80(5):125. doi: 10.1007/s00018-023-04763-2 (PMC10115737; doi:10.1007/s00018-023-04763-2)
Supplement: Supplementary file 1 — Supplementary file1 (DOCX 414 KB) [file 18_2023_4763_MOESM1_ESM.docx]

**Supplementary Information**

**
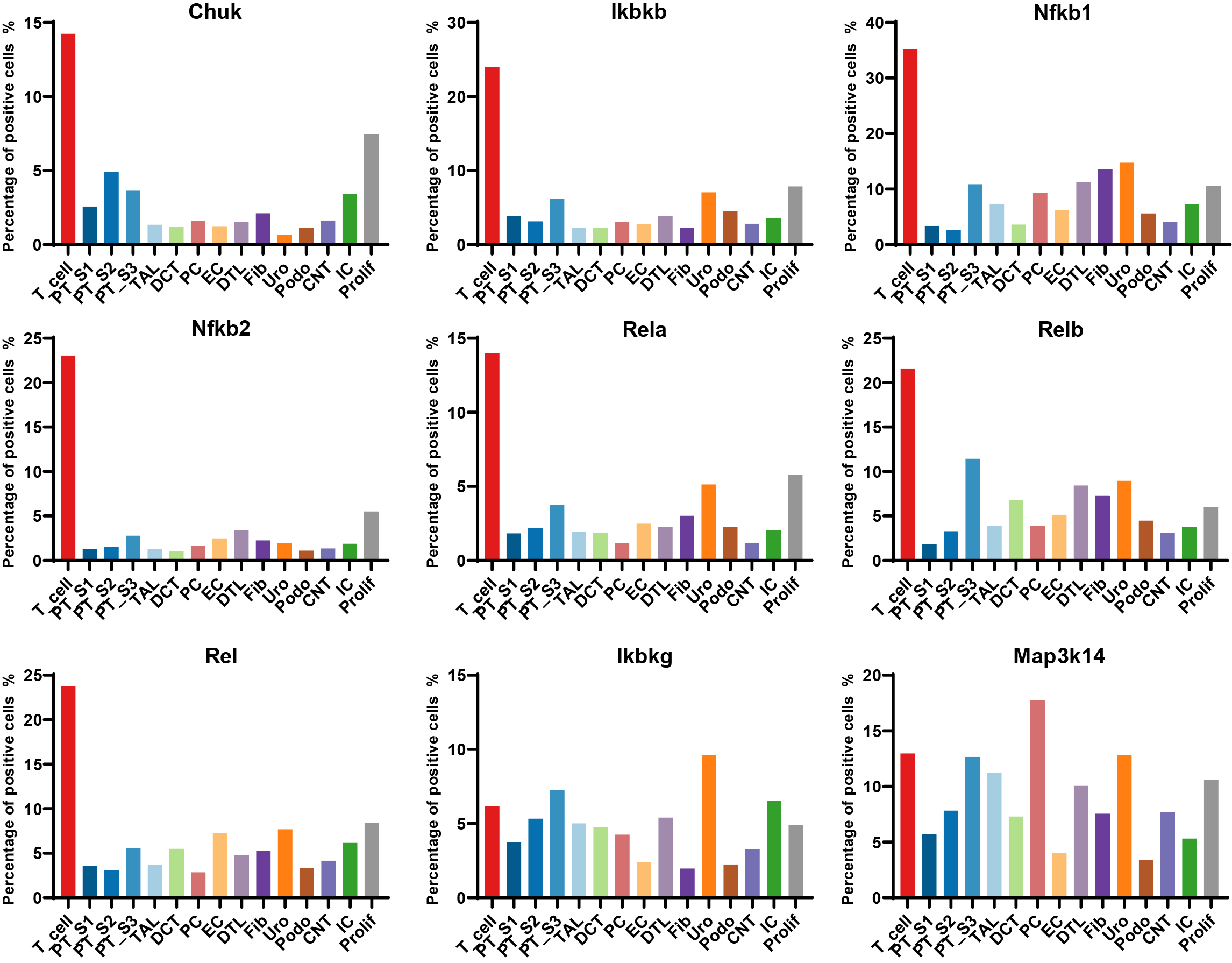
**

**Fig. S1: Proportion of NFkB pathway related gene positive cells in different cell types in kidneys after IRI-induction at day 2.**

**
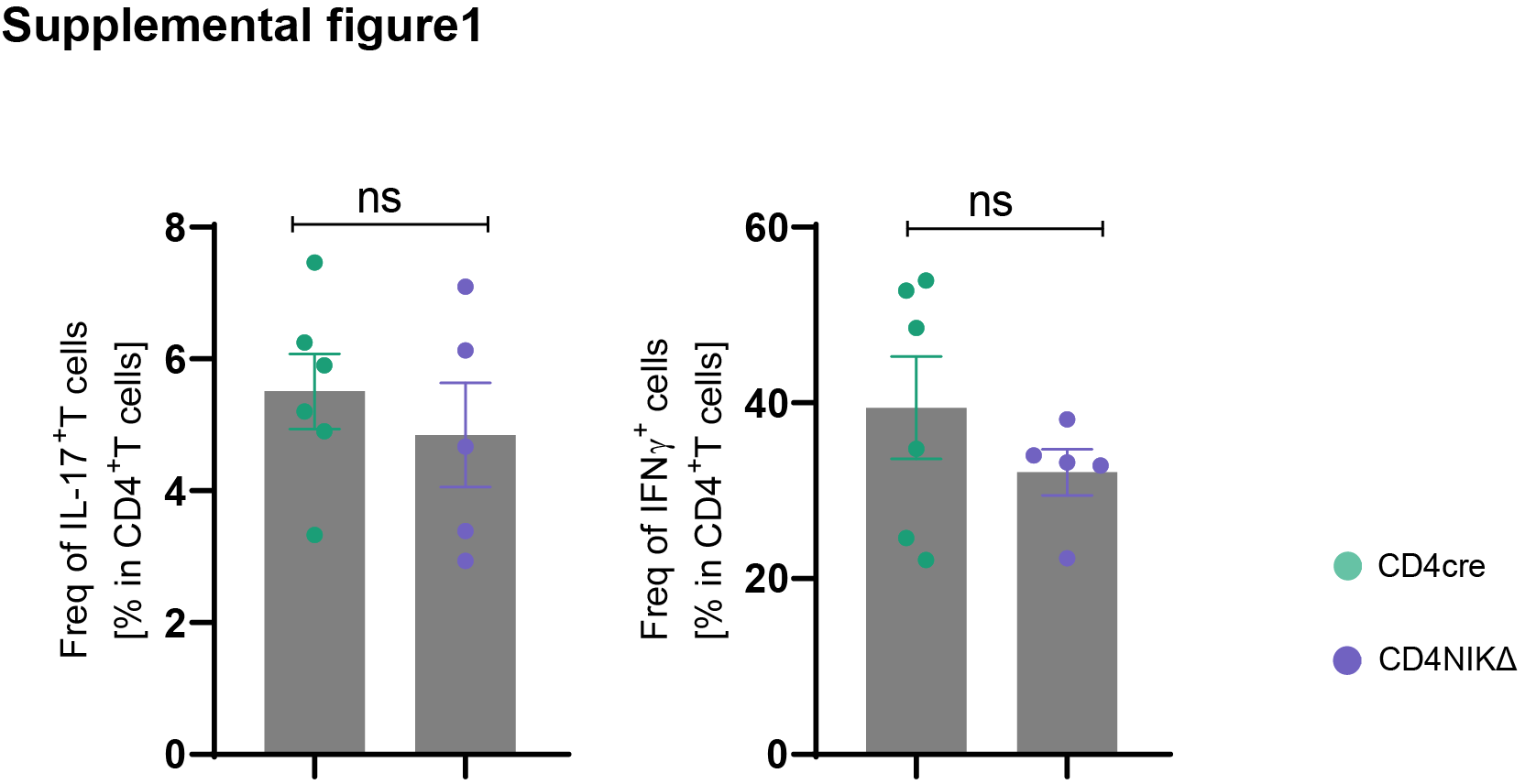
**

**Fig. S2: Deficiency of NIK in CD4^+^ T cells has no effect on cytokines production during IRI .**

Quantification of indicated cytokines produced in renal CD4^+^T cells from CD4cre and CD4NIKΔ mice. Symbols represent individual data points with the mean as a bar.

**
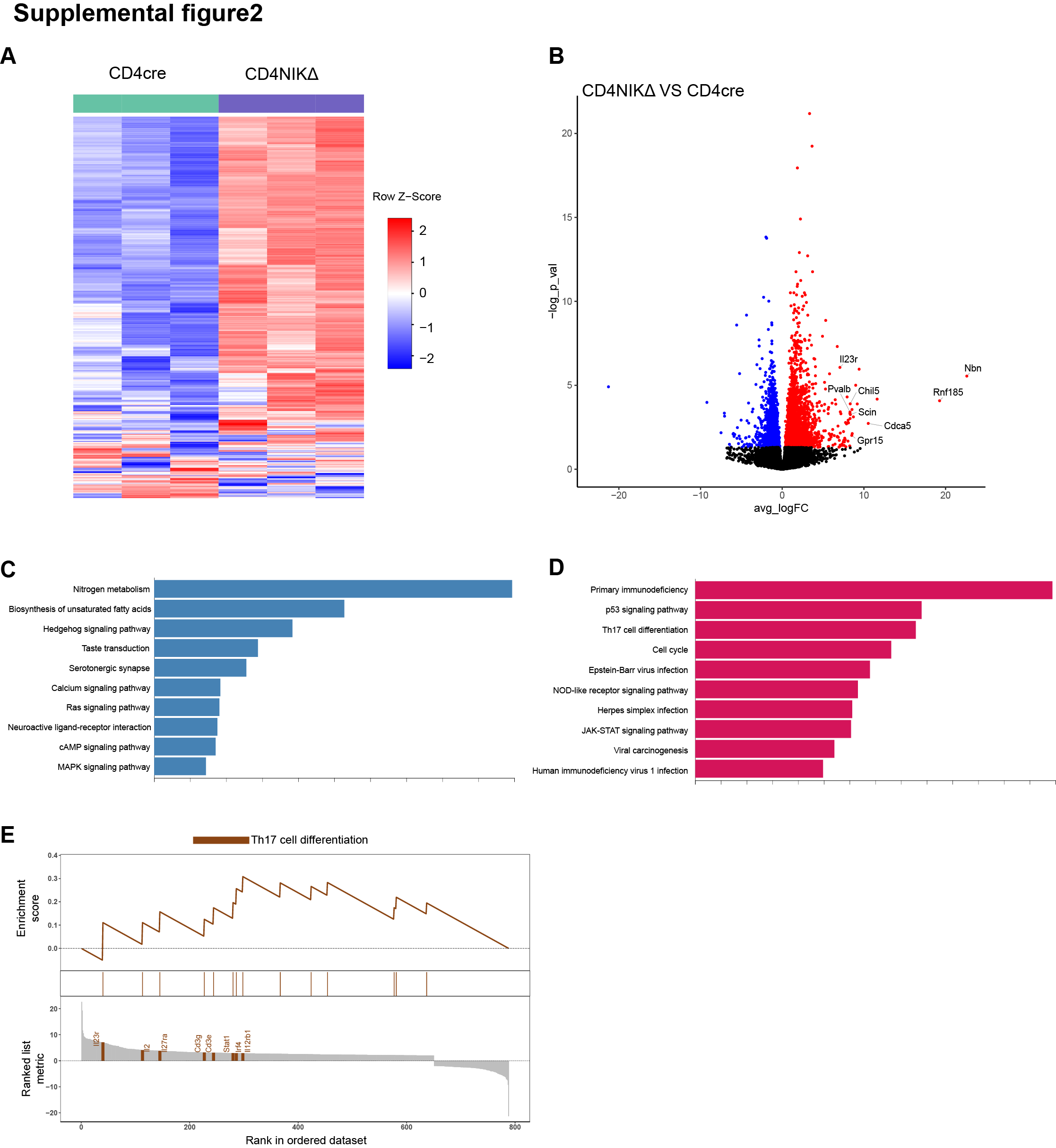
**

**Fig. S3: NIK has no effect on the pathogenicity of CD4^+^ T cells during IRI.**

**(A)** Heatmap shows 200 random genes expressed by renal CD4^+^ T cells from CD4cre (N=3) and CD4NIK1Δ (N=3) mice on day 2 after IRI. **(B)** Volcano plot shows differentially expressed genes (DEG) between CD4^+^ T cells from CD4cre and CD4NIKΔ (blue: downregulated in CD4NIKΔ; red: upregulated in CD4NIKΔ; black: adjusted p value less than 0.05). **(C)** Bar plot shows top 10 enriched KEGG pathways of downregulated genes in CD4^+^ T cells of CD4NIKΔ mice. **(D)** Bar plot shows top 10 enriched KEGG pathways of upregulated genes in CD4^+^ T cells of CD4NIKΔ mice. **(E)** GSEA enrichment plot shows enriched genes of Th17 differentiation pathway.
